# Visual intelligence for efficient human action recognition in human computers interaction applications

**DOI:** 10.1371/journal.pone.0343132

**Published:** 2026-03-05

**Authors:** Noorah Alghasham, Waleed Albattah

**Affiliations:** Department of Information Technology, College of Computer, Qassim University, Buraydah, Saudi Arabia; Institute of Management Sciences Peshawar, PAKISTAN

## Abstract

Human Action Recognition (HAR) is a pivotal area in computer vision, video surveillance, and human-computer interaction (HCI), driven by the need for efficient and accurate models to enhance HCI experiences. Traditional HAR methods often rely on hand-crafted features and shallow learning techniques, which limits their ability to capture complex patterns. In contrast, this study proposes an efficient HAR model that leverages deep neural networks, specifically a combination of Convolutional Neural Networks (CNNs) and Recurrent Neural Networks (RNNs), to enhance HCI through AI-powered action understanding. The model employs a pre-trained EfficientNetB7 network to extract rich spatial features from video frames, followed by a Long Short-Term Memory (LSTM) network to capture long-range temporal dependencies. This architecture enhances recognition accuracy while reducing computational complexity, making it highly suitable for HCI applications. Experimental results demonstrate the superior performance of the model, achieving a classification accuracy of 97.8% on the UCF101 dataset and 80.1% on the HMDB51 dataset, outperforming state-of-the-art HAR models. The proposed model eliminates the need for auxiliary assistive techniques like data augmentation, highlighting its efficiency and tremendous potential for real-world HCI applications that rely on accurate and efficient recognition of human actions.

## 1. Introduction and background

### 1.1 Overview

Human Action Recognition (HAR) has recently become a significant and active research area within the computer vision field and has a powerful effect on many real-world applications. Human actions are analyzed for various real-world applications, including behavior analysis, abnormal activity detection, video retrieval, human-computer interaction, healthcare, education, entertainment, surveillance, and security [1,2].

HAR is recognized as a video classification problem, which aims to analyze and understand the human performed action such as physical activity, in a segmented video consisting of only a single action, and then assign a specifically defined label to the action. Human actions range from simple movement of an arm or leg to a complex integrated movement of multiple body parts of arms, legs, and head [3]. However, video-based HAR classification is more challenging and complicated than static image classification. Video-based HAR classification involves classifying various actions from a series of frames, considering that the action could not be performed throughout the video. Therefore, HAR is a complicated process that requires modeling long-term temporal information in addition to spatial information [4]. Additionally, the real-world application is an uncontrolled environment where human actions are diverse and complex, thus making HAR process a more challenging task [5].

Recently, with the fast advancement of deep learning methods, HAR studies applied deep learning methods since they are end-to-end trainable methods that automatically extract abstract features from input data. Deep learning methods such as Convolutional Neural Networks (CNNs) and Recurrent Neural Networks (RNNs) are very efficient at learning complicated actions because of their properties of local dependency and scale invariance.

Many deep learning-based HAR models have been improved and achieved superior performance. The success of deep learning-based HAR models in accurately recognizing human actions is based on their high computational cost. Precisely, they adopt different techniques to improve the accuracy and robustness of the HAR models, such as applying different temporal and spatial data augmentation techniques, leveraging multiple input data modalities, i.e., the fusion of RGB, structure, and depth data modalities [6], or adopting additional features such as hand-crafted iDTs [7]. The research community has focused on achieving high-level classification accuracy despite the complexity; consequently, current efficient HAR models remain complex and consumes a lot of time and resources, such as CPU, GPU, and energy consumption [6].

However, despite this significant improvement, building an efficient and fast deep learning-based HAR model remains essential as reducing computational cost and resource consumption is still an open research issue.

Based on the aforementioned problem, this study aims to build an efficient deep learning-based HAR model that utilizes a CNN and RNN to recognize complex actions with minimal computation cost, without the need for auxiliary techniques such as data augmentation techniques or utilizing multimodal input data.

The proposed model implements recurrent layers, such as Long Short-Term Memory (LSTM), to process sequential data besides CNN layers, which process matrix-like spatial data. Specifically, it relies on a pre-trained EfficientNetB7 [8] to learn the spatial features of video frames and then applies LSTM atop 2D ConvNets to learn long-range temporal information from the extracted spatial features.

### 1.2 Human action

Human action is a simple or complex, interactive movement of one or more human body parts. The most commonly used alternative keywords for human action include activity, or behavior [2].

Human actions can be divided into four levels based on body parts involved in the action and the complexity of the action [9]. Following is the description of each human action level.

- **Gesture is** a simple movement of the hand, face, or other body parts that represents a specific meaning or message—for example, facial expressions, a hand wave, etc.
- **Actions are** Several physical movements -gestures- are carried out by one person, such as walking, running, and swimming.
- **Interaction** is an action or set of actions carried out by two people. One of the actors is human, whereas the other can be a human or object. Therefore, it is classified as human-to-object and human-to-human interactions. For example, shaking hands and hugging are human-to-human interactions, and applying lipstick and knitting are human-to-object interactions.
- **Group Activity** is the most complex type of human activity. It involves a set of gestures, actions, and interactions performed by more than two people and may include objects. Examples of group activities include playing volleyball, studying together, soccer games, and others [2,5,10].

Human action can be performed at various speeds, illumination levels, and viewpoints [2]. However, several human actions appear similar in videos, such as walking and running. Therefore, the similarity between action classes poses a significant challenge for the HAR model. Furthermore, when different individuals perform the same type of action, the classification task becomes increasingly difficult [11].

### 1.3 Vision-based HAR Methods

Vision-based HAR Methods have been extensively applied due to the availability of visual data and their advantages [5]. Visual data representing human actions includes RGB color, 3D structure, depth, infrared sequences, point clouds, and event streams. RGB data formats, such as 2D images and 3D videos, are the most commonly used data types in the HAR model, which are typically easy to compile and contain detailed information about the appearance and context of the captured scene. One of the most prominent examples of RGB sensors is in closed-circuit television (CCTV) systems and cameras that track and record human activity. Thus, their real-world application is to provide surveillance and safety in public places. The performance of RGB vision-based HAR models is often challenging, and it mainly depends on image quality, which is influenced by different factors, such as image resolution, background variations, lighting environments, and illumination changes [3]. Additionally, RGB videos typically have larger data sizes, resulting in high computational costs for modeling the spatio-temporal information for HAR models [6].

**Fig 1:**
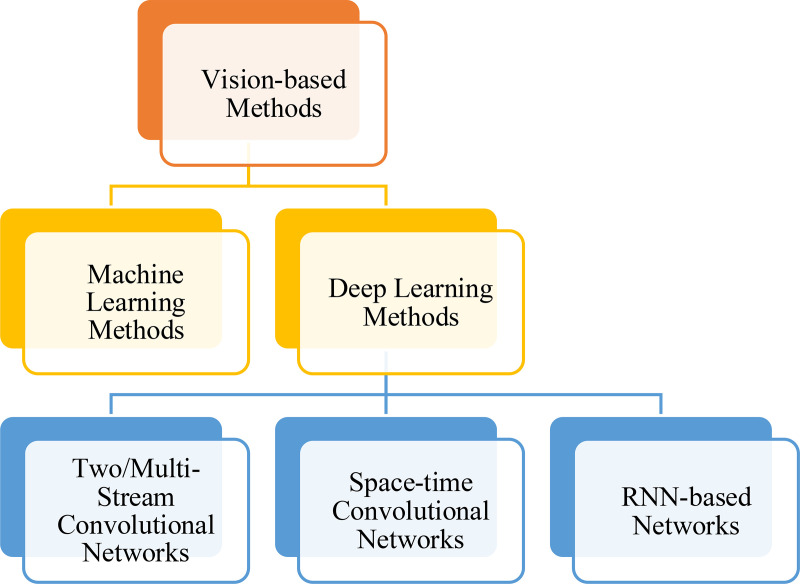
Different types of vision-based HAR methods.

Vision-based HAR methods may be classified as machine learning techniques applied to hand-crafted features using machine learning methods or end-to-end deep learning methods [3]. Fig 1 illustrates different types of vision-based HAR methods.

#### 1.3.1 Deep learning-based methods.

DL Methods have been proposed to overcome the existing limitations of ML methods, including hand-crafted and domain-specific features with added advantages. First, DL methods can handle large, interconnected or unstructured datasets. Additionally, they can learn from unlabeled data, which is helpful in unsupervised and reinforcement learning [5,10]. Second, the most significant aspect of DL methods is an end-to-end trainable model that automatically extracts abstract features from input data, for example, image sequences, without requiring specialized knowledge and significant effort [10,12]. Table 1 shows a brief comparison between ML-based HAR and DL-based HAR with some advantages and disadvantages [4,10].

**Table 1:** Advantages and disadvantages of vision-based HAR Methods.

Vision-based HAR Methods	Advantages	Disadvantages
Machine Learning-based HAR	**−** Perform efficiently with small training data. **−** Consume limited memory and computing time. **−** The model training by using known features.	**−** Required pre-processing and normalizing of the input data to enhance performance. **−** Extracting features manually. **−** Required features selection and dimensionality reduction. **−** Unable to deal with complicated actions.
Deep Learning based HAR	**−** Does not need data pre-processing and normalization. **−** Does not require expert knowledge to extract suitable features. **−** Automatically learned abstract features from raw data. **−** Can handle complex task by extract high-level representation with deep layers.	**−** Required large-scale dataset to prevent overfitting. **−** Complex and consume high computation time and memory usage.

Accordingly, DL-based HAR models may be classified into three main categories based on the DL network architectures and how they extract spatial features and model temporal information from the videos: two-stream/multi-stream convolutional networks, spatio-temporal convolutional networks, and RNN-based networks. Table 2 summarizes the DL-based HAR models and discusses the significant advantages and limitations of each category.

**Table 2:** Summary of different types of DL-based HAR models.

DL-Based HAR Models	Structure	Advantages and significant limitations
A Two/Multi-Stream Convolutional Networks	Consists of two 2D CNN streams as backbone models, RGB stream and optical flow stream, for learning spatial-related and temporal-related information representing the performed action.	− Although using an optical flow stream improves providing the HAR model with short-term movement information, it needs to capture the temporal information with long-range dependencies. − Requiring pre-computing optical flow stacks and storing them on the local storage. It is time-consuming, requires massive storage capacity, and is not an end-to-end trainable model.
Space-time Convolutional Networks	Use 3D convolution networks to model video motion information instead of using optical flow multi-frame stacks as input. 3D convolution structure consists of two dimensions for spatial information, i.e., height and width, and the third dimension for temporal information [1].	− 3D convolution network has more parameters and is complex; thus, it is hard to train and improve. − Training a 3D convolution network on a large-scale dataset is time-consuming and requires high computation. **−** Additionally, the temporal features are unable to extract long-term temporal dependencies.
RNN-based Networks	Is implemented recurrent layers, for instance, Long Short-Term Memory (LSTM) or Gated Recurrent Units (GRU), atop the CNN layers to learn both spatial appearance and temporal motion features of human action. Specifically, it used LSTM to process sequential data besides CNN, that process matrix-like spatial data.	− Unlike the mentioned methods to capture temporal-related information, LSTM is capable of capturing long-term temporal information. Accordingly, RNN-based networks proved their efficiency in the HAR system by successfully learning spatial motion information and temporal sequencing with long-range dependencies

### 1.4 Related work

The most important work on HAR combines CNN and RNN as a powerful unified model. Table 3 provides a brief comparison between them. For instance, [13] proposed a real-time HAR framework for industrial surveillance by using CNN-based saliency detection to extract human-relevant frames and FlowNet2 optical flow to capture motion cues. A multi-layer LSTM then models temporal dynamics for action classification. The method achieved 94.9% on UCF50, 94.45% on UCF101, 72.21% on HMDB51, 95.8% on YouTube Actions, and 69.5% on Hollywood2. While effective for surveillance applications, its dependence on optical flow significantly increases computational cost and limits end-to-end efficiency.

**Table 3:** Various RNN-based HAR studies.

Study	Used Method	Dataset	Performance (Accuracy %)	Input Data Modality	The Used Transfer Learning Architecture
[13]	MobileNet + FlowNet2 + Multi-Layer LSTM	UCF-50 UCF-101 HMDB-51 YouTube Actions Hollywood2	94.9% 94.45% 72.21% 95.8% 69.5%	RGB + Optical flow input frames	A pretrained MobileNet + FlowNet2
[14]	VGG-16 + single-layer LSTM with 320 hidden units	UCF-11	91.94%	RGB frames	A pre-trained VGG16
[16]	LSTM + global memory (MobileNetv2)	FCVID ActivityNet	78.6% 69.5%	RGB frames	A pre-trained MobileNetv2
[17]	ResNet50 and ResNet101 + ConvLSTM + spatio-temporal attention mechanism	UCF-101 HMDB-51	87.11% 53.07%	RGB frames	A pre-trained ResNet50 and ResNet101
[18]	GoogleNet and ResNet + attention mechanism + temporal coherence analysis + convolutional LSTM	UCF-11 UCF-101 HMDB-51	93.48% 92.8% 67.1%	RGB + Optical flow input frames	A pre-trained GoogleNet + ResNet
[21]	VGG-16 + deep autoencoder (DAE) + a quadratic SVM	UCF-50 UCF-101 HMDB-51 YouTube action	96.4% 94.33% 70.3% 96.21%	RGB frames	A pre-trained VGG16
[22]	Inception V3 + Kalman filter + MobileNet + GRU	Cornell Activity Dataset (CAD-60)	95.5%	RGB-D data: RGB + skeleton	A pre-trained Inception V3 + MobileNet
[23]	VGG-16 + C2LSTM + SoftMax classifier	UCF-101 HMDB51	92.8% 61.3%	RGB frames	A pre-trained VGG16
[24]	p-non-local module with VGG-19 + Fusion KeyLess Attention with 3 layers Bi-LSTM	HMDB51 Hollywood2	50.1% 59.6%	RGB frames	A pre-trained VGG19
[25]	YOLO3 + MOSSE tracker + LiteFlowNet + DS-GRU	UCF-50 UCF-101 HMDB51 Hollywood2 YouTube Actions	95.2% 95.5% 72.3% 71.3% 97.17%	RGB + Optical flow input frames	A pre-trained YOLO3 + MOSSE tracker + LiteFlowNet
[29]	Spurious-3D Residual Network (s-ResNet3D) + Two-Level Attention Module (Frame Attention + Spatial Attention)	UCF101 HMDB51	95.68% 72.6%	RGB frames	s-ResNet3D (trained from scratch, not pre-trained)
[30]	Fusion of Multiview handcrafted features + Deep features extracted from CNN + Feature selection (Entropy + MI + SCC) + Naïve Bayes classifier	HMDB51 KTH UCF-Sports YouTube IXMAS	93.7% 97% 98% 99.4% 95.2%	RGB frames (multi-view video data)	A pre-trained VGG19
[31]	CNN + ConvLSTM + LRCN (CNN + LSTM)	UCF50 HMDB51	CNN: 99.58% ConvLSTM: 81.97% LRCN: 93.44% CNN: 92.70% ConvLSTM: 67.67% LRCN: 71.55%	RGB frames	All models trained from scratch
[32]	Multilevel feature extraction + Global Average Pooling + Bidirectional GRU + Scaled Dot-Product Attention + Grad-CAM visualization	UCF101 HMDB51	98.13% 81.45%	RGB frames + optical flow + spatial saliency maps + motion saliency maps	A pre-trained InceptionV3

Likewise, [14] proposed a combined CNN-LSTM model for video activity recognition. The propped model extracts 40 frames of each input video and applies a pre-trained VGG-16 network [15] to capture spatial features from every frame. Then, a single LSTM layer containing 320 hidden units was used to model temporal features. Finally, a dense layer classified the video into a particular class. The experiment was conducted on the UCF11 dataset and achieved a success rate of 91.94%. Nevertheless, training small datasets may require a more robust model with large and complex datasets.

Moreover, [16] introduced AdaFrame, an adaptive action-recognition framework that reduces computational cost by processing only the most informative video frames. It uses a memory-augmented LSTM guided by a global memory module, with features extracted via a pre-trained MobileNetV2, to dynamically select relevant frames. Evaluated on FCVID and ActivityNet, AdaFrame achieved 78.6% and 69.5% mAP while requiring only 4.92 and 3.8 frames per video, demonstrating the effectiveness of adaptive frame selection for efficient video understanding.

A spatio-temporal attention mechanism was introduced in [17] for video action recognition. The model employs a pre-trained CNN including ResNet50 and ResNet101 to generate spatial saliency masks, while a convolutional LSTM captures temporal attention by identifying informative frames. Additional regularization strategies were proposed to enhance attention localization. Using only RGB input and applying data augmentation, the model achieved accuracies of 87.11% on UCF101 and 53.07% on HMDB51.

Furthermore, the work [18] developed an attention-driven ConvLSTM framework that identifies salient spatiotemporal regions in video sequences. Spatial features are extracted using pre-trained GoogleNet [19] and ResNet [20] models, then refined through an attention module combining LSTM with a spatial transformer network to emphasize informative feature maps. A temporal coherence mechanism suppresses redundant frames, while convolutional LSTMs capture spatial–temporal dependencies for action classification. The model achieved accuracies of 93.48% on UCF11, 92.8% on UCF101, and 67.1% on HMDB51 using RGB and optical flow inputs, demonstrating the effectiveness of attention-guided spatiotemporal modeling for HAR.

The work [21] introduced a real-time HAR framework designed for online video streams captured under unstable network and environmental conditions. The system extracts spatial features using a pre-trained VGG-16 network, then applies a Deep Autoencoder (DAE) to model temporal variations while reducing feature dimensionality. A quadratic SVM classifier is subsequently used to categorize compressed features into action labels, processing video in 15-frame segments with continuous fine-tuning based on incoming data streams. The model achieved accuracies of 96.4% on UCF50, 94.33% on UCF101, 70.3% on HMDB51, and 96.21% on YouTube Actions. Its key contribution lies in enabling real-time deployment in unstable environments, making it suitable for surveillance and healthcare monitoring systems.

Extending this direction, [22] proposed a multi-feature HAR framework that integrates visual, temporal, and 2D skeleton information to improve action recognition performance. The system employs a pre-trained MobileNet model [22] for 2D skeleton extraction, while InceptionV3 is used to capture visual appearance features and track human motion. Temporal dynamics are further modeled using a Kalman filter, and the fused features are finally classified using a GRU network. The model was evaluated on the CAD-60 dataset, comprising indoor RGB-D activity sequences, and achieved an overall accuracy of 95.5%, demonstrating strong capability in recognizing human actions from RGB data.

A redesigned LSTM architecture, termed Correlational Convolutional LSTM (C2LSTM), was proposed by [23] to jointly learn spatial features, motion cues, and temporal dependencies from video sequences. The model integrates convolution operations within LSTM units for spatial extraction and correlation operators to capture motion between consecutive frames. Using pre-trained VGG-16 for spatial feature extraction and a SoftMax classifier for prediction, the system achieved accuracies of 92.8% on UCF101 and 61.3% on HMDB51. Spatiotemporal data augmentation was applied to reduce overfitting, and the results indicate competitive performance relative to RGB-only and optical-flow-based methods.

Moreover, the work [24] developed a hybrid convolutional and recurrent model for video motion recognition. They proposed a novel p-non-local module with CNN to learn long-distance dependencies and reduce computation. They then adopted Fusion KeyLess Attention with bidirectional LSTM to model the sequential patterns of the interesting part of human motion. The model was implemented using a pre-trained VGG-19 as a CNN network with three layers of Bi-LSTM and only RGB input data. Thus, the best accuracies are 50.1% for HMDB51 and 59.6% for Hollywood2.

In parallel, [25] developed a lightweight real-time HAR framework based on a dual-gate recurrent unit (DS-GRU), a fast recurrent architecture that uses only two gates and no memory cell. The system includes human detection via YOLOv3 [26], followed by tracking using the MOSSE algorithm [27]. Motion features are extracted from pairs of consecutive frames using the optical-flow-based LiteFlowNet [28], and temporal dynamics are modeled through a five-layer DS-GRU network with skip connections. The model was evaluated on UCF50, UCF101, HMDB51, Hollywood2, and YouTube Actions, achieving classification accuracies of 95.2%, 95.5%, 72.3%, 71.3%, and 97.17%, respectively. The study highlights the efficiency of DS-GRU for real-time applications while maintaining competitive recognition performance.

Moreover, [29] proposed a human action recognition model built on a spurious-3D residual network enhanced with a two-level attention mechanism. The architecture integrates frame-level and spatial attention modules to strengthen temporal and spatial feature representation from RGB video inputs. The model was evaluated on the UCF101 and HMDB51 benchmark datasets, achieving 95.68% accuracy on UCF101 and 72.60% on HMDB51, demonstrating improved recognition performance compared to several baseline deep learning approaches.

The work in [30] proposed a HAR framework that fuses multiview handcrafted features with deep features extracted using a pre-trained VGG19 network. Horizontal gradients, vertical gradients, and directional descriptors were combined with CNN-based representations, followed by feature selection using entropy, mutual information, and SCC, and final classification via a Naïve Bayes model. Experiments on HMDB51, KTH, UCF Sports, YouTube, and IXMAS achieved accuracies of 93.7%, 97%, 98%, 99.4%, and 95.2%, respectively. However, the approach is not an end-to-end deep learning model, as it relies on separate handcrafted and deep feature extraction stages, which limits its ability to learn spatiotemporal representations automatically.

Additionally, [31] investigated three deep learning architectures for HAR using RGB video inputs: a CNN for spatial learning, a ConvLSTM for spatiotemporal modeling, and an LRCN combining CNN and LSTM features. Performance was assessed on the UCF50 and HMDB51 datasets, where the CNN achieved the highest accuracies of 99.58% and 92.70%, followed by LRCN with 93.44% and 71.55%, and ConvLSTM with 81.97% and 67.67%. Despite the strong performance of the CNN, its reliance on frame-level spatial cues limits its ability to capture temporal information. Furthermore, all three models were trained from scratch without transfer learning, which increases computational cost and reduces generalization capability.

More recently, [32] proposed a multilevel human action recognition framework that integrates RGB frames, optical flow, spatial saliency, and motion saliency to construct a comprehensive spatiotemporal representation. Each modality is processed using a pre-trained InceptionV3 model followed by Global Average Pooling, and the fused features are modeled using a Bidirectional GRU with a scaled dot-product attention mechanism. The model demonstrated strong performance on standard HAR benchmarks, achieving 98.13% accuracy on UCF101 and 81.45% on HMDB51, surpassing several state-of-the-art deep learning approaches. These results highlight the effectiveness of combining multilevel features with temporal sequence modeling for robust action recognition.

Thus far, as previously mentioned, recent related video-based HAR studies have been conducted using different deep-learning methods. While these have proven effective in recognizing human actions, they are complex, require intensive computations, and consumes significant amounts of time and resources. Furthermore, researchers have applied different data augmentation techniques, pre-trained CNN models, multi-modal input data, and the adoption of hand-crafted features such as iDTs, among others. Given the complexity of the related work, a computationally efficient HAR model that can run for real-world applications remains needed. Therefore, this study aims to build an efficient and lightweight HAR model with minimal computational overhead.

## 2. Human action recognition research methodology

**Fig 2:**
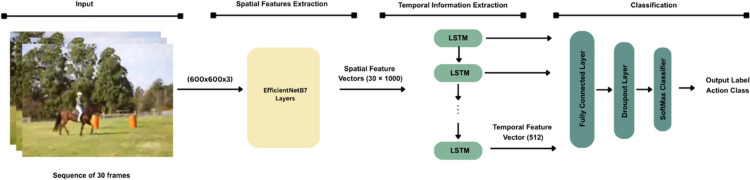
The proposed HAR framework.

The proposed methodology focuses on human action recognition in real-world applications and uncontrolled environments using the HMDB51 and UCF101 datasets. The methodology consists of the following steps: data preparation, and building DL-based HAR model, including convolutional and recurrent layers. Fig 2 illustrates the general framework of the proposed model.

### 2.1 Data preparation

**Fig 3:**
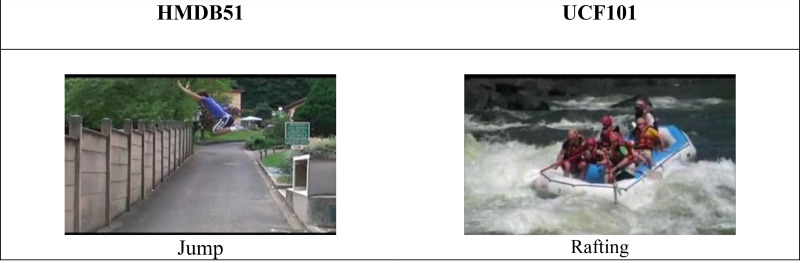
Sample frames of HMDB51 and UCF101.

The proposed model was trained and evaluated on two most commonly used benchmark datasets, based on RGB video, namely HMDB51 [33] and UCF101 [34]. Thus, the data were obtained by downloading the datasets from their official websites. The datasets used are realistic, containing real-world action videos obtained from diverse conditions. Using a realistic dataset is challenging due to several limitations, such as cluttered background, viewing angle variations, illumination variations, and occlusions [5]. Table 4 summarizes the significant aspects of the datasets and further details used about the datasets used are provided in section 3. Additionally, Fig 3 shows examples of action class frames from the HMDB51 and UCF101 datasets.

**Table 4:** Summary of the used datasets.

Aspect/Dataset	HMDB51	UCF101
Year	2011	2012
Classes/Actions	51	101
No. of Videos	6,849	13,320
Resolution	320 X 240	320 X 240
Frame Rate	30 fps	25 fps
Input- modality	RGB	RGB
No. of Actors	Unspecific	Unspecific
Type of View	Single	Single

Based on the characteristics of the datasets used, it is clear that the two datasets used, UCF101 and HMDB51, have been cleaned, segmented, and annotated. This means that each video in the dataset contains only one human action performed, which should be classified into a specific action category. Furthermore, end-to-end trainable deep learning architectures can be trained directly from unprocessed data, such as raw pixels [35].

Therefore, preparing these raw videos for training requires special data preprocessing techniques. The essential preprocessing techniques include frame/clip sampling and image resizing to a uniform size, scaling, and label encoding.

A. **Video Clips Constructing**

Since a video represents an action through a sequence of frames with varying lengths, many techniques have been developed to convert these videos into different clips of similar lengths to build an effective classification model [6]. Thus, the proposed model generates video clips by extracting every 30 frames as a fixed-length video segment, forming a new sequence of fixed-length video segments.

Afterward, the clip frames are resized to a fixed height and width acceptable in the proposed CNN structures; thus, the proposed model converts frames to a size of 600 x 600. Finally, it converts these frames to NumPy arrays.

B. **Scaling**

Scaling is used to rescale the raw input data to a specific value range, because deep learning methods are more efficient at training on small input values, such as the range from 0 to 1 [12]. Thus, scaling converts pixel values to the range [0, 1] or [−1, 1] for image input data. Scaling is used to avoid large weight values that lead to high computational costs and overflow on computers [36].

Every deep learning model requires a specific type of input preprocessing; therefore, for this HAR model, EfficientNet convolution layers were applied, and input data was required in the format of float pixel values in the range 0–255. Furthermore, the input preprocessing method participates in the EfficientNet architecture as a ***Rescaling layer.*** Thus, this model accepts input images of the shape (600, 600, 3), and their pixel values should range [0, 255] while scaling is included as part of the model.

C. **Label Encoding**

Action Labels are typically categorical data, such as eating, pushing, cycling, diving, etc. Furthermore, deep learning methods cannot work correctly on categorical data; therefore, all input data must be converted to numerical data. However, label encoding is essential to encode each label as an integer. Specifically, One-Hot encoding is a powerful method that recognizes the natural ordering relationships of classes. It is an identity array whose size is equal to the number of action classes. Therefore, each row represents a specific action that contains only one element with the value of 1 to identify the action [12]. Therefore, the proposed model applied One-Hot encoding to convert these categorical class labels into binary classes.

### 2.2 Deep learning model

The proposed HAR model is an RNN-based deep learning model for recognizing human actions based on RGB video. It is an integrated model containing convolutional layers that learn spatial appearance features, as well as recurrent layers to learn temporal motion information and long-range dependencies.

#### 2.2.1 Convolutional neural network.

Convolutional Neural Networks (CNNs) are among the most widely used deep learning models for visual recognition tasks due to their strong capability in extracting hierarchical spatial features. A CNN consists of an input layer, multiple hidden layers, and an output layer. The hidden layers typically include convolutional layers, which apply learnable kernel filters to capture local patterns; pooling layers, which perform non-linear down-sampling to reduce spatial dimensionality and model complexity; and fully connected layers, which integrate the extracted features for final classification. Because of their effectiveness in handling 2D grid-structured data, CNNs are extensively used in HAR systems to extract spatial representations from video frames before temporal modeling [10,37,38].

Moreover, using pre-trained CNN architectures, VGG [15], ResNets [39], EfficientNet [8], significantly reduces the cost of training deep models from scratch and improves computational efficiency. Through transfer learning, these models can be fine-tuned to perform effectively on smaller datasets while mitigating challenges such as overfitting and inadequate regularization.

In this study, EfficientNetB7 [8] was employed as the spatial feature extractor due to its strong performance on ImageNet and its effectiveness as a pre-trained classification model. The extracted spatial feature maps are then used as input to the subsequent temporal modeling stage.

EfficientNet is a family of convolutional models (B0–B7) designed to achieve an optimal balance between accuracy and computational efficiency through a unified Compound Scaling method, which uniformly increases network depth, width, and resolution. EfficientNetB0 serves as the mobile-size baseline discovered via neural architecture search (NAS), while the larger variants, including EfficientNetB7, are scaled versions of this baseline and consistently outperform many state-of-the-art CNNs on the ImageNet benchmark [8].

In this study, EfficientNetB7 was selected as the spatial backbone due to its high representational capacity and strong transfer learning performance. Its ability to capture fine-grained spatial patterns makes it particularly suitable for Human Action Recognition (HAR), where subtle appearance variations across frames must be accurately modeled. The spatial features extracted by EfficientNetB7 subsequently serve as input to the temporal modeling stage, enabling effective recognition of complex actions.

The architecture of the EfficientNetB0 baseline model is illustrated in Fig 5. Since they are scaled, the architectures of EfficientNet B0 to B7 are similar but different in resolution, channels, and layer numbers. Table 5 explains the fundamental differences between all EfficientNet models [8,40].

**Table 5:** Differences between EfficientNet-B0 to EfficientNet-B7.

Base Model	Resolution	No. of parameters	Advantages	Disadvantages
EfficientNetB0	224	5.3M	Lightweight, low computational cost, ideal for mobile and embedded devices.	Lower accuracy, limited feature extraction, not ideal for complex datasets.
EfficientNetB1	240	7.8M	Higher accuracy with moderate efficiency, suitable for real-time applications.	Higher computational requirements, still limited for large-scale applications.
EfficientNetB2	260	9.2M	Balanced trade-off between efficiency and performance, robust to variability.	Increased computational load, limited gains over B1, higher power consumption.
EfficientNetB3	300	12M	Better accuracy while keeping computation reasonable, handles complex features.	Requires more memory, not ideal for real-time applications on low-power devices.
EfficientNetB4	380	19M	High accuracy, effective for high-resolution images, good efficiency balance.	Requires better hardware to operate efficiently.
EfficientNetB5	456	30M	Very high accuracy, beneficial for critical applications, advanced feature extraction.	High computational and memory requirements, not ideal for resource-constrained devices.
EfficientNetB6	528	43M	Top-tier accuracy, excellent for high-precision tasks, handles intricate patterns.	High computational and memory demands, needs specialized hardware.
EfficientNetB7	600	66M	Highest accuracy in the EfficientNet family, best for extremely detailed analysis.	Extremely high computational and memory requirements, impractical for real-time applications.

**Fig 4:**
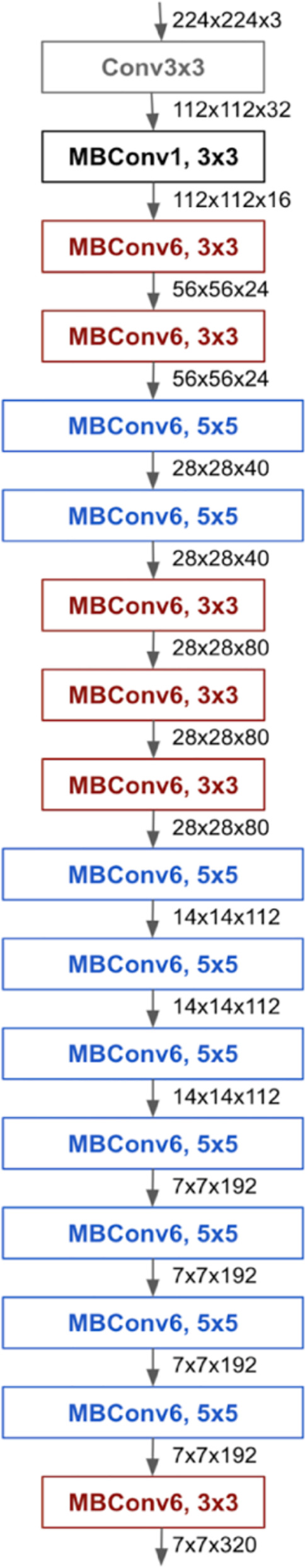
EfficientNetB0 baseline architecture.

After successfully extracting the 2D spatial features maps of videos’ frames, they passed to the following LSTM layers to model the temporal relationships and motion information with long-range dependencies (Fig 4).

#### 2.2.2 Recurrent neural network.

An RNN is a recurrent neural network for sequential data. While CNNs are more efficient at dealing with 2D grid-like data such as images and video streams, RNN is more capable of processing sequential data like speech, text, and video streams. An RNN uses the outputs of a layer as inputs for a new layer to predict the outputs of the current layer. Because RNN uses hidden state features to memorize prior inputs, RNN considers both the current and previously received inputs for every time step [12].

**Fig 5:**
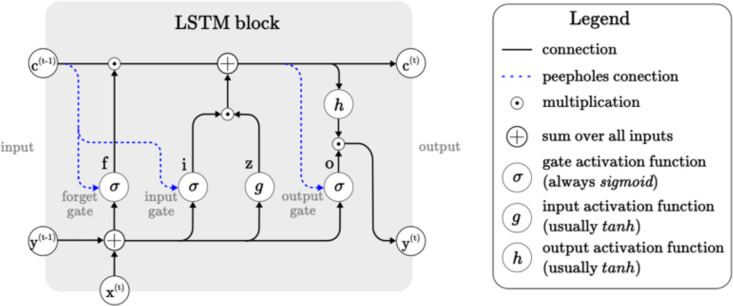
Architecture of an ideal LSTM cell [43].

Although RNNs are specially developed to deal with sequential data and outperform other general neural networks, they suffer from the problems of vanishing or exploding gradients. Vanishing or exploding gradients limit the memory of the recurrent connections and prevent the network from adequately learning long-term temporal dependencies. Therefore, RNN-LSTM is the most common method to avoid the problem of vanishing and exploding gradients [41]. LSTM is an enhanced version of RNN that can learn long-term sequential dependencies. LSTM structures replace hidden units with memory cells, whose inputs and outputs are controlled by gates to store or forget features extracted from prior time steps. A typical LSTM cell contains input, forget, and output gates, as well as a cell activation component, as illustrated in Fig 5 [42,43].

The proposed model integrates EfficientNetB7 as a spatial feature extractor with a lightweight LSTM module for temporal modeling. Each video frame is first processed through EfficientNetB7, and the resulting 1,000-dimensional feature vectors are arranged sequentially using a TimeDistributed layer to preserve temporal order. This produces a feature sequence that serves as input to the recurrent stage.

To efficiently capture long-term temporal dependencies, the model employs a single LSTM layer with 512 hidden units. This design avoids the computational overhead associated with deeper LSTM architectures while retaining the ability to model motion dynamics across frames. The LSTM output is then passed through a fully connected layer with 512 neurons and ReLU activation, followed by a dropout rate of 0.5 to reduce overfitting.

Finally, a SoftMax classification layer generates the predicted action label. This streamlined CNN–LSTM configuration provides an effective balance between accuracy and computational efficiency, making it suitable for real-time and resource-constrained HAR applications. Table 6 shows a summary of the proposed model.

**Table 6:** The proposed CNN-LSTM model summary.

Model: “sequential”		
Layer (type)	Output Shape	Param #
time_distributed (TimeDistributed)	(None, 30, 1000)	66658687
lstm (LSTM)	(None, 512)	3098624
dense (Dense)	(None, 512)	262656
dropout (Dropout)	(None, 512)	0
dense_1 (Dense)	(None, 101)	51813
Total params: 70,071,780 Trainable params: 69,761,053 Non-trainable params: 310,727		
Model Created successfully!		

## 3. Datasets

Benchmark datasets are essential for evaluating Human Action Recognition (HAR) models. While early datasets were collected in controlled environments with limited variability, recent real-world RGB video datasets, such as UCF101 and HMDB51, capture diverse challenges including background clutter, camera motion, and illumination changes. These realistic datasets provide a more reliable basis for assessing the robustness of deep learning–based HAR systems [3,5,11].

- **HMDB51**

Human Motion Data Base (HMDB51) published by the Serre research laboratory at Brown University, contains videos from different sources, including YouTube, Google videos, movies, and the Prelinger archive. HMDB51 contains 6849 manually labeled videos to represent daily human actions and is categorized into 51 action classes, each including at least 101 clips. Action classes can be divided into five groups: general facial actions such as smiling and talking; facial actions with object manipulation like eating and smoking; general body movements such as walking and waving; body movements with object interaction such as brushing hair and riding a bike, and body movements for human interaction like; hug and punch. Besides action labels, meta-labels are provided for each clip, including information like video quality, background, camera motion, lighting conditions, visible body, and the number of people participating in the action. Thus, the dataset is considered challenging because of a wide variation in scenes, camera viewpoints, and the cluttered background of real-world videos [4,5].

- **UCF101**

UCF101 is an extension of the UCF50 dataset published by the computer vision laboratory at the University of Central Florida. UCF101 contains 13,320 realistic videos gathered from YouTube in “.avi” format and classified into 101 action classes. The number of videos for all classes is balanced, ranging from 100 to 130, with a 2–7-second duration for each video clip. Since it is a realistic dataset, i.e., a real-life action video dataset, its videos vary considerably in camera viewpoint and movement, background, lighting, and the number of objects. Thus, videos of each action class are grouped into 25 groups, each with 4–7 videos sharing some features, such as the background. The 101 action classes are divided into five types: Human-Object Interaction, which has 20 classes; Body-Motion Only which has 16 classes; Human-Human Interaction which includes five classes; Playing Musical Instruments that has ten classes; and Sports which has 50 classes [4,37].

## 4. Experiments and results

### 4.1 Experimental setting

This section explains the experimental setting and the implementation details of the proposed deep learning methods. First, the proposed deep learning model was implemented using Python programming language. *Python* is a high-level, general-purpose programming language commonly utilized to build machine learning algorithms. Additionally, Python provides many libraries like TensorFlow and Keras API to develop deep neural networks [44].

Thus, the proposed model was implemented and assessed utilizing Python with Anaconda package management. Specifically, it uses TensorFlow 2.7.0 and Keras 2.7.0 on top of TensorFlow for easily building and training neural network models. These essential tools and libraries were installed on a computer with NVIDIA GeForce RTX 3080 GPU with 32 GB RAM and ran on the local GPU.

Furthermore, the proposed CNN and LSTM deep learning model is trained through Adam optimizer because of its ability to update the weights adaptively, with a learning rate of 0.001, and calculate loss by the categorical cross-entropy loss function. The training data is trained in mini-batches of 128 size and 100 iterations for learning patterns in the sequential data. The model’s performance was evaluated at the end of every iteration, i.e., training epoch, on validation data with 128 batch size.

### 4.2 Results and discussions

After the HAR model has been developed and compiled, the proposed model is fitted to the dataset. UCF101 and HMDB51 datasets are utilized to train the model. Besides, each dataset was typically split, based on experiments, into 90% for training and 10% for testing to test the model’s performance after each iteration and prevent overfitting.

The integrated CNN and LSTM model was trained and evaluated for human action recognition. Many experiments have been conducted with different hyperparameter settings to find the optimal model. Thus, the proposed model is assessed utilizing several accuracy calculation metrics, such as confusion matrix, precision, recall, and the F- score.

**Fig 6:**
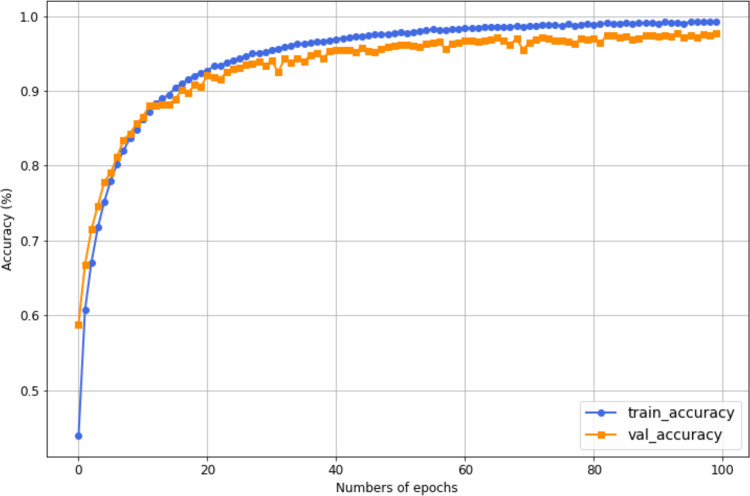
Accuracy curves of training and validation for the most accurate HAR model on UCF101, where training accuracy is 0.99, and the validation accuracy is 0.98.

The training and validation accuracy curves for the most accurate proposed HAR model on UCF101 are presented in Fig 6. As shown in the figure, a significant improvement in both training and validation accuracies is observed as the number of epochs increases. The results indicate that after an initial phase of rapid learning, both metrics converge and stabilize, with minimal fluctuations in validation accuracy. The small gap between the two accuracies suggests limited overfitting, indicating that the model generalizes well to unseen data. Overall, the training process is effective, with both training and validation accuracies reaching high, stable values in the later epochs. The training accuracy reached 99.21%, while the validation accuracy achieved 97.77%.

**Fig 7:**
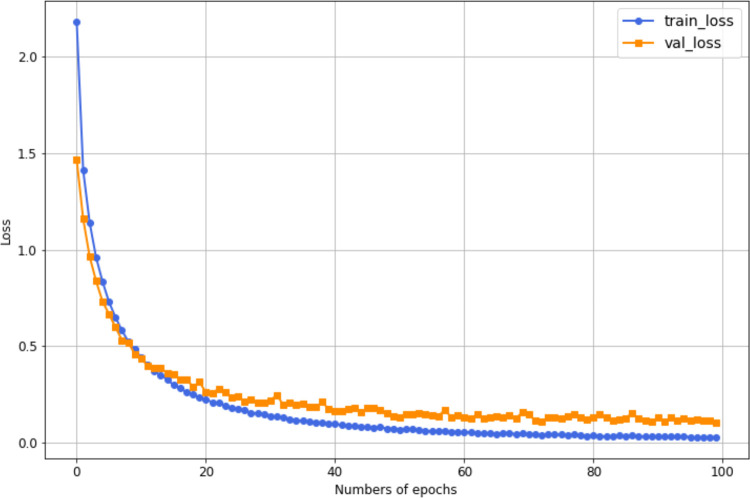
Loss curves of training and validation for the most accurate HAR model on UCF101, where the training loss is 0.03, and the validation loss is 0.1.

However, Fig 7 shows the training and validation loss curves for the proposed HAR model on UCF101. As illustrated in the figure, training, and validation losses decrease significantly as the number of epochs increases. During the early epochs, the model experiences rapid improvement, with both losses declining steeply. Both losses gradually stabilize as training progresses, with the training loss slightly lower than the validation loss throughout the later epochs. This slight gap between the two curves indicates minimal overfitting, suggesting the model maintains its ability to generalize to unseen data. Overall, the training process appears effective, as both losses continue to decrease and stabilize in the later epochs, reflecting an improvement in the model’s performance.

**Fig 8:**
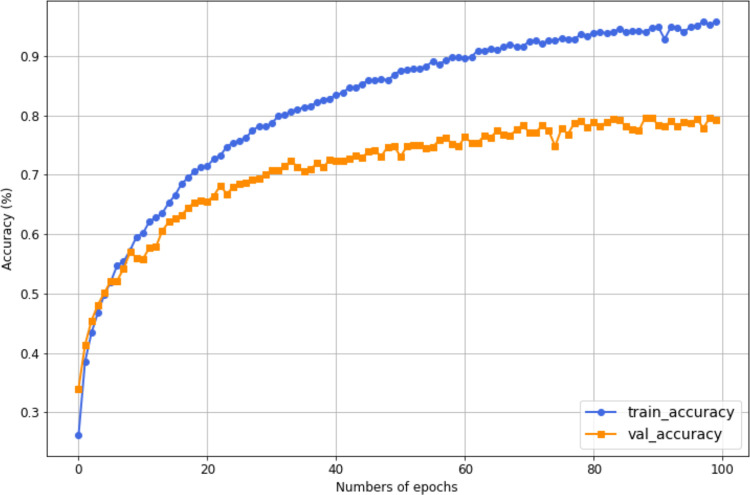
Accuracy curves of training and validation for the most accurate HAR model on HMDB51, where training accuracy is 0.96, and the validation accuracy is 0.80.

Moreover, Fig 8 shows the training and validation accuracy curves on HMDB51. The training accuracy increases consistently, reaching approximately 95.83%, while the validation accuracy improves steadily, leveling off at around 80.08%. This pattern suggests that the model learns effectively from the training data and generalizes reasonably well to unseen data. The relatively stable gap between the two curves indicates that the model maintains strong performance on both the training and validation sets, reflecting a balanced learning process. While there is a difference between training and validation accuracy, the model shows consistent improvement across both metrics over time, highlighting its capacity to handle the dataset with reasonable accuracy.

**Fig 9:**
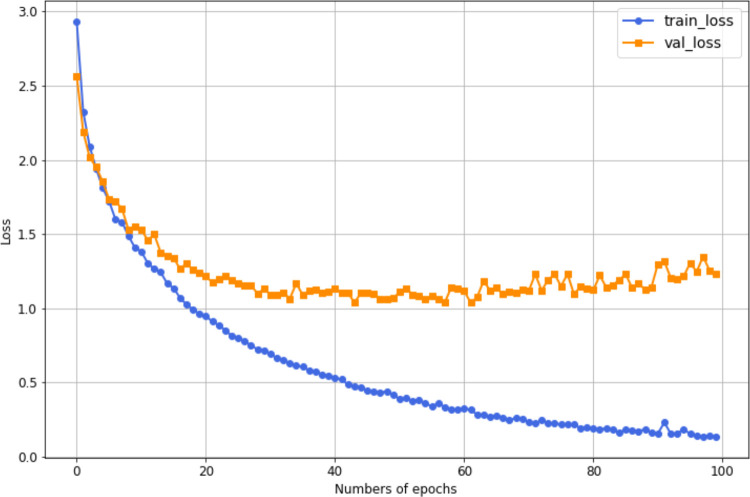
Loss curves of training and validation for the most accurate HAR model on HMDB51, where training loss is 0.13, and the validation loss is 1.2.

Additionally, Fig 9 shows the training and validation loss curves over several epochs. The training loss decreases consistently, indicating that the model learns effectively from the training data. In contrast, the validation loss decreases initially but then stabilizes around the 60th epoch at 1.2, suggesting that the model’s ability to generalize to unseen data is limited. The difference between the two curves indicates that while the model performs well on the training data, it may not generalize as effectively to the validation data.

**Fig 10:**
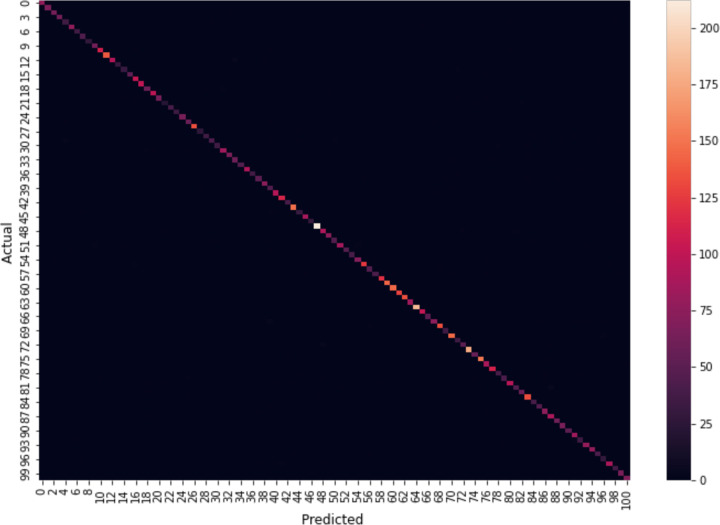
Confusion matrix of UCF101 dataset.

The confusion matrix for the testing data on the UCF101 dataset, as shown in Fig 10, illustrates a clear diagonal pattern. This indicates that most of the predictions made by the model were correct, with most predicted classes aligning with the true classes (represented by the diagonal). The intensity of the colors along the diagonal suggests that the model achieved high accuracy in classifying the data. However, there are a few off-diagonal points, which indicate instances where the model misclassified certain samples. The color bar on the right indicates the scale of values, with lighter shades representing higher values (more correct predictions) and darker shades representing fewer instances. Overall, the matrix suggests a strong model performance with minimal misclassifications.

**Fig 11:**
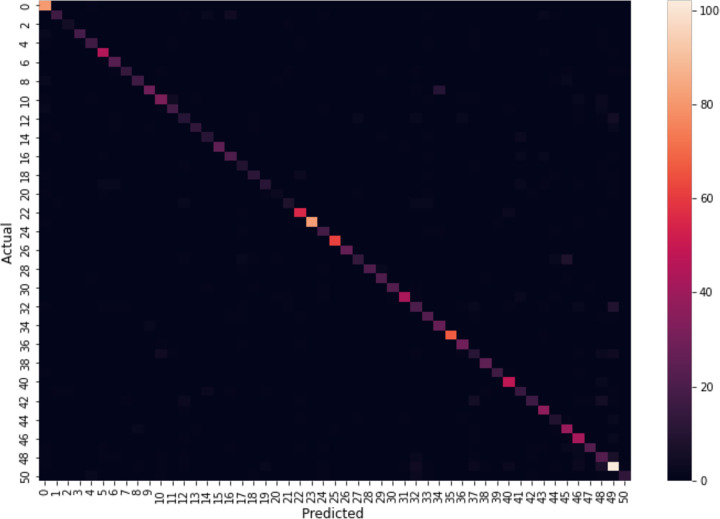
Confusion matrix of HMDB51 dataset.

Moreover, the confusion matrix for the testing data on the HMDB51 dataset is shown in Fig 11. The effectiveness of the proposed model is demonstrated by the strong intensity along the diagonal, representing the true positives for each class, which is consistently high across all classes. This indicates that the model performs well in correctly classifying most of the test data for each category. The results in terms of accuracy, precision, recall, and F1-score for both UCF101 and HMDB51 are summarized in Table 7.

**Table 7:** The efficiency of the proposed model using different performance metrics.

Metric\Datasets	UCF 101	HMDB 51
Training accuracy	0.9921	0.9583
Testing accuracy	0.9777	0.8008
Precision	0.9777	0.8008
Recall	0.9773	0.7926
F1-score	0.9772	0.7899

The proposed model achieved the highest testing accuracy of 0.9777, besides the positive prediction score of 0.9777, sensitivity score of 0.9773, and F1-score of 0.9772. These statistics show the efficiency of the proposed HAR model on the UCF101 dataset.

Furthermore, the HAR model achieved a competitive testing accuracy of 0.8008 on the HMDB51 dataset, which is significantly better than other state-of-the-art HAR models, as seen in Table 3. The proposed model has achieved true positive, sensitivity, and effectiveness scores of 0.8008, 0.7926, and 0.7899, respectively. These results are because HMDB51 is a more challenging dataset since video clips collected from various subjects with different illumination and viewpoint changes perform the same action, thus resulting in more significant errors during training and testing.

However, the experiments indicate that many factors could affect the performance of the proposed deep learning model, including choosing the appropriate hyper-parameters settings, the appropriate learning rate on network convergence, etc.

Many experiments have been conducted with different hyper-parameters settings, network layer types, the number of hidden units, batch size, loss function, etc., to prevent overfitting and achieve higher classification accuracy. Thus, a practical model that recognizes human actions accurately must be built.

However, besides applying the EfficientNetB7 for spatial feature extraction, a simple structure of the LSTM model, consisting of a single layer with 512 hidden units, has been used for long-range temporal information learning. The choice of LSTM structure, such as the number and types of layers, i.e., vanilla or bi-directional, adding dropout, etc., are determined experimentally.

### 4.3 Comparison with state-of-the-art studies

This experiment marks a significant advancement in Human Action Recognition (HAR), contributing valuable insights to the development of human behavior detection systems. To assess the performance and efficiency of the proposed model, its classification accuracy and parameter number were compared against state-of-the-art HAR models, as illustrated in Table 8. The proposed model achieved superior recognition performance while maintaining significantly lower computational cost compared with existing HAR approaches. As shown in Table 7, earlier studies such as [13] and [25] relied on two-stream architectures that combine optical flow with RGB frames—an approach that enhances temporal information but requires pre-computing, storing, and loading optical flow, making these models computationally expensive and unsuitable for end-to-end learning. Similarly, [30] depended on RGB multiview inputs and extensive handcrafted–deep feature fusion to enhance accuracy, which further increases model complexity.

**Table 8:** Comparison with State-of-the-art HAR Studies.

Study	Performance (Accuracy %)		Complexity
	UCF 101	HMDB 51	Number of Parameter (M)
[13]	94.45%	72.21%	Not available
[21]	94.33%	70.3%	>138
[25]	95.5%	72.3%	7.29
[29]	95.68%	72.6%	30.12
[30]	–	93.7%	144
[31]	–	71.55%	Not available
[32]	98.13%	81.45%	28.3
The proposed Model	97.77%	80.08%	70

In contrast, the proposed method achieves 97.8% on UCF101 and 80.1% on HMDB51 using RGB inputs only, thereby eliminating the need for pre-computed motion data and reducing storage and processing overhead. The lightweight LSTM configuration—with a single layer of 512 hidden units—further enhances computational efficiency and enables the model to capture long-range temporal dependencies effectively, making it well suited for real-time applications. Although [32] reported slightly higher accuracy (98.13% on UCF101 and 81.45% on HMDB51), their performance was achieved through multiple input modalities, including optical flow, spatial saliency maps, and motion saliency maps, which substantially increase computational complexity compared with the streamlined RGB-only design of the proposed model.

To ensure a comprehensive assessment of computational complexity, Table 8 summarizes the number of parameters for all benchmarked models. The proposed model utilizes 70M parameters, positioning it between lightweight and high-capacity architectures. Several prior methods exhibit much greater complexity—such as [30] with 144M parameters and [21] with more than 138M parameters—whereas the parameter values for [13] and [31] were not reported. In contrast, lighter models, including [25] with 7.29M, [32] with 28.3M, and [29] with 30.12M, achieve reduced complexity by relying on additional modalities such as optical flow, multiview inputs, or saliency-based representations. The proposed model therefore offers a more favorable trade-off, delivering strong recognition performance with a moderate parameter size.

The proposed model introduces key contributions that distinguish it from existing deep learning–based HAR approaches. Its primary innovation lies in the integration of EfficientNetB7 with an LSTM module, enabling efficient extraction of both spatial and temporal features within a unified framework. This design enhances classification accuracy while eliminating the need for auxiliary components—such as optical flow computation or extensive data augmentation—that are typically computationally expensive and resource-intensive.

Empirical results further validate the robustness and adaptability of the model under challenging real-world conditions, including variations in illumination, complex backgrounds, and diverse camera viewpoints. Consequently, the proposed framework demonstrates strong applicability across multiple domains, such as intelligent surveillance, human–computer interaction, and security systems.

Compared with contemporary models that rely on optical flow or multimodal inputs, the proposed approach offers clear advantages in computational efficiency and deployment simplicity. Overall, the model represents a meaningful advancement in deep learning–based human action recognition, achieving an effective balance between performance and computational cost.

## 5. Conclusion and future work

Human Action Recognition (HAR) has become increasingly important in computer vision and intelligent video surveillance, particularly with the rapid advancements in deep learning. Although many state-of-the-art HAR models achieve high accuracy, they often rely on computationally expensive components—such as optical flow, multimodal inputs, or complex fusion strategies—which limit their practicality for real-time and resource-constrained environments.

This study introduced an efficient deep learning–based HAR model that integrates EfficientNetB7 for spatial representation learning with an LSTM module for modeling long-range temporal dependencies. By relying solely on RGB frames and eliminating the need for pre-computed motion features or data augmentation, the proposed architecture significantly reduces computational overhead while preserving strong discriminative capability.

Experimental results demonstrate that the model achieves 97.77% accuracy on UCF101 and 80.08% on the challenging HMDB51 dataset, outperforming several state-of-the-art methods. Additional evaluation metrics—including precision (97.77%), sensitivity (97.73%), and F1-score (97.72%) on UCF101, and competitive performance on HMDB51—further confirm the robustness and reliability of the proposed approach. The lower performance on HMDB51 is attributed to inherent dataset complexity, including variations in scene illumination, viewpoints, and actor appearance. Overall, the results validate the efficiency and effectiveness of the proposed HAR model, highlighting its suitability for real-world deployments in surveillance, security, and human–computer interaction systems, where both accuracy and computational efficiency are essential.

Several promising research directions emerge from this study. First, extending the model to handle multi-person interactions or overlapping actions would enhance its applicability to crowded or dynamic environments. Second, advancing the model toward online or continuous action recognition for untrimmed video streams is a critical step for real-time monitoring systems. Furthermore, future research may explore developing lightweight HAR models optimized for resource-limited and mobile devices such as smartphones and wearable sensors. Although the computational capabilities of such devices continue to improve, running deep HAR models efficiently on-device remains an open challenge. Designing architectures that maintain high accuracy while operating under strict resource constraints represents a significant opportunity for future advancements.
